# Activation of neuronal *N*-methyl-d-aspartate receptor plays a pivotal role in Japanese encephalitis virus-induced neuronal cell damage

**DOI:** 10.1186/s12974-018-1280-8

**Published:** 2018-08-25

**Authors:** Zheng Chen, Xugang Wang, Usama Ashraf, Bohan Zheng, Jing Ye, Dengyuan Zhou, Hao Zhang, Yunfeng Song, Huanchun Chen, Shuhong Zhao, Shengbo Cao

**Affiliations:** 1 State Key Laboratory of Agricultural Microbiology, Huazhong Agricultural University, Wuhan, Hubei 430070 People’s Republic of China; 2 Key Laboratory of Preventive Veterinary Medicine in Hubei Province, College of Veterinary Medicine, Huazhong Agricultural University, Wuhan, Hubei 430070 People’s Republic of China; 3 The Cooperative Innovation Center for Sustainable Pig Production, Huazhong Agricultural University, Wuhan, Hubei People’s Republic of China; 4 Key Laboratory of Agricultural Animal Genetics, Breeding, and Reproduction of the Ministry of Education, Huazhong Agricultural University, Wuhan, China

**Keywords:** JEV, NMDAR, Activation, MK-801, Neuronal death

## Abstract

**Background:**

Overstimulation of glutamate receptors, especially neuronal *N*-methyl-d-aspartate receptor (NMDAR), mediates excitatory neurotoxicity in multiple neurodegenerative diseases. However, the role of NMDAR in the regulation of Japanese encephalitis virus (JEV)-mediated neuropathogenesis remains undisclosed. The primary objective of this study was to understand the function of NMDAR to JEV-induced neuronal cell damage and inflammation in the central nervous system.

**Methods:**

The effect of JEV-induced NMDAR activation on the progression of Japanese encephalitis was evaluated using the primary mouse neuron/glia cultures and a mouse model of JEV infection. A high-affinity NMDAR antagonist MK-801 was employed to block the activity of NMDAR both in vitro and in vivo. The subsequent impact of NMDAR blockade was assessed by examining the neuronal cell death, glutamate and inflammatory cytokine production, and JEV-induced mice mortality.

**Results:**

JEV infection enhanced the activity of NMDAR which eventually led to increased neuronal cell damage. The data obtained from our in vitro and in vivo assays demonstrated that NMDAR blockade significantly abrogated the neuronal cell death and inflammatory response triggered by JEV infection. Moreover, administration of NMDAR antagonist protected the mice from JEV-induced lethality.

**Conclusion:**

NMDAR plays an imperative role in regulating the JEV-induced neuronal cell damage and neuroinflammation. Thus, NMDAR targeting may constitute a captivating approach to rein in Japanese encephalitis.

## Background

Japanese encephalitis virus (JEV)—a mosquito-borne flavivirus—belongs to the family *Flaviviridae*, which also includes some other important human pathogens such as West Nile, yellow fever, Zika, St. Louis encephalitis, and hepatitis C viruses. JEV-associated illness, referred as Japanese encephalitis (JE), predominantly occurs in most countries of South and East Asia and the Western Pacific with roughly 68,000 cases annually, half of which occurs in China [1]. About 20 to 30% of clinical JE cases are fatal, and 30 to 50% of the survivors suffer from persistent neurological sequelae. Even those JE patients with seemingly good recovery display some incessant neurological deficits [2].

During infection, JEV breaches the blood-brain barrier and subsequently invades the central nervous system (CNS), wherein virus replication induces a colossal inflammatory response and consequently causes neuronal cell death [3]. The decisive factor in JEV-induced neuroinflammation is the unbridled release of neuronal toxicity factors and inflammatory cytokines/chemokines such as tumor necrosis factor–α (TNF-α), interleukin-1β (IL-1β), IL-6, chemokine (C-C motif) ligand 5 (CCL5, also called RANTES), and monocyte chemotactic protein 1 (MCP1, also called CCL2) [4–6]. These inflammatory and neuronal toxicity factors endorse massive leukocyte incursion into the brain and promote the death of both infected and non-infected neurons [4]. During JEV infection, neuronal cells may also undergo necrosis and apoptosis as the result of virus replication [7, 8]. Further studies underlying the molecular mechanisms of JEV-induced neuronal death are still required.

Glutamate is a major excitatory neurotransmitter in the CNS and plays a pivotal role during the neurodegenerative process [9–11]. Glutamate-mediated neurotoxicity is a pathogenic event which is shared by several brain disorders. The neuronal cell damage induced by glutamate involves an aberrant Ca^2+^ influx mainly mediated by the *N*-methyl-d-aspartate receptor (NMDAR) which belongs to the ionotropic class of glutamate receptors [12–14]. Subsequently, the Ca^2+^ inflow causes the activation of calpains and some other protease-mediating cytoskeleton damage responses, accompanied by reactive oxygen species production, mitochondrial dysfunction, and eventually neuronal cell apoptosis [14–18]. Moreover, a recent study demonstrated the involvement of PCK/ERK pathway in regulating the NMDAR-mediated neurotoxicity [19]. Interestingly, it has also been reported that JEV-activated microglial cells release glutamate, which causes neurotoxicity and eventually leads to neuronal cell death [20]. However, the role of glutamate receptors in JEV-mediated pathogenesis is still undefined.

In the present study, we found that JEV infection enhances the phosphorylation of neuronal NMDAR, which in turn contributes to neuronal cell damage. Our in vitro and in vivo data revealed that blockade of NMDAR by its antagonist (MK-801) reduces neuroinflammation and prevents neuronal death caused by JEV. These results suggest that neuronal NMDAR plays an important role in JEV-induced encephalitis and that NMDAR may serve as a putative target for JE therapy.

## Methods

### Cell cultures and virus

Neuron/glia cultures were prepared from the cerebral cortices of embryonic day 15 C57BL6J wild-type mice. The recent isotropic fractionator and histological data demonstrate a neuron to glia ratio of 1:1 in the human brain, and this ratio is considered as constant across the brain structures and species, so the proportion of neurons in neuron/glia cultures is usual around 50% [21–23]. The primary cells were seeded on polylysine-coated (20 mg/mL) dishes at a density of 10^5^ cells per well in Dulbecco’s modified Eagle’s medium (DMEM) supplemented with 5% fetal bovine serum. At 6 h (h) post-seeding, the culture medium was replaced with neurobasal medium supplemented with 2% B-27, 0.5% streptomycin and penicillin, and 0.5 mM L-Glu. The cells were used for subsequent experiments after incubation for 7 days. N2a cells (mouse neuroblastoma cell line) were cultured and maintained in DMEM that was supplemented with 10% fetal bovine serum, 100 U/ml penicillin, and 100 mg/ml streptomycin sulfate. Non-adherent cells were removed by washing with non-supplemented DMEM prior to further treatment. The neuron/glia or N2a cell cultures were mock- or JEV-infected at the multiplicity of infection (MOI) of 1. After 1 h for virus absorption, the cells were treated with MK-801 or DMSO at indicated concentrations.

The JEV wild-type strain P3 used in this study was propagated in suckling mouse brain and BHK-21 cells for in vivo and in vitro experiments, respectively.

### JEV infection and antagonist administration

Adult 6-week-old BALB/c mice were purchased from the Laboratory Animal Center of Huazhong Agricultural University, Wuhan, China. Mice were randomly assigned to four groups: group 1, control group (DMSO; *n* = 15); group 2, only MK-801-treated group (MK-801; *n* = 15); group 3, JEV-infected group (JEV; *n* = 15); and group 4, JEV- and MK-801-treated group (JEV + MK-801; *n* = 15). Mice belonging to the JEV and JEV + MK-801 groups were intraperitoneally injected with 10^6^ PFU of the JEV P3 strain in 200 μl of DMEM [24, 25]. MK-801 (10 mg/kg body weight per mouse) was intravenously administered in mice belonging to the JEV + MK-801 group on days 3 and 4 post-infection. Mice in the DMSO group received DMSO and those in the MK-801 group received MK-801. On day 5 post-infection, mice infected with JEV developed signs of acute viral encephalitis. Five mice in each group were euthanized, and brain samples were collected for further studies. The remaining 10 mice in each group were monitored daily to assess behavior and mortality.

### Cell viability assay

The viability of cultured cells was detected using the CellTiter-Glo® One Solution Assay kit (Promega) according to the procedure supplied by the manufacturer. Primary neurons/glia were seeded in 96-well white opaque plates at a density of 1 × 10^5^ cells/ml. After incubation for 24 h at 37 °C with 5% CO_2_, the culture supernatants were replaced with different concentrations of MK-801 or DMSO, and each concentration was tested in triplicates. The cells were incubated with MK-801 or DMSO for 2 h. Subsequently, the treated cells were washed with phosphate-buffered saline (PBS) for three times and incubated with fresh medium for 72 h. To determine the cell viability, the cells were washed with PBS, and 100 μl of the CellTiter-Glo reagent was added to each well. The cells were subjected to agitation for 2 min on a shaker for appropriate cell lysis. Later, the plates were incubated at room temperature for 10 min. The luminescence value was read by a multimode plate reader. The quantification of luminescence signals in each condition was compared with its corresponding DMSO control.

### Immunofluorescence assay

Primary mouse neuron/glia cultures were infected with JEV and treated with MK-801, then cells were fixed in 4% formaldehyde for 10 min and blocked with 10% bovine serum albumin (BSA) in PBS for 30 min. Afterward, cells were stained with rabbit polyclonal anti-caspase-3 antibody (Proteintech) and mouse monoclonal anti-neuronal nuclei (NeuN) antibody (Chemicon) for 1 h. Then cells were washed three times with PBS and incubated with Alexa Fluor 488- and 555-conjugated secondary antibody (Invitrogen) for 30 min. The cell nuclei were stained with DAPI (Invitrogen). The staining results were observed using a fluorescence microscope (Zeiss).

### Enzyme-linked immunosorbent assay (ELISA)

The cell culture supernatants were collected from treated cells at the time points mentioned in the figure legends and stored at − 80 °C. The mice brain tissues were homogenized in DMEM and stored at − 80 °C. Before performing ELISA, the mice brain homogenates were thawed and centrifuges at 5000×*g*, and the supernatants were collected. The protein levels of extracellular glutamate and inflammatory cytokines (TNF-α, CCL-5, IL-1β, and CCL-2) in the supernatants of cultured cells or centrifuged-brain lysates were determined by ELISA kits according to the manufacturer’s instructions. TNF-α, CCL-5, IL-1β, and CCL-2 ELISA kits were purchased from eBioscience. The Mouse Glu ELISA kit for the detection of glutamate was obtained from X-Y Biotechnology.

### RNA extraction and quantitative real-time PCR

Total RNA was extracted using TRIzol reagent (Invitrogen), and 1 μg of RNA was used to synthesize cDNA using a first-strand cDNA synthesis kit (Toyobo). Quantitative real-time PCR was performed using a 7500 real-time PCR system (Applied Biosystems) and SYBR green PCR master mix (Toyobo). The relative expression of TNF-α, CCL-5, IL-1β, and CCL-2 were normalized to the levels of endogenous control β-actin within each sample using the 2^−ΔΔ*CT*^ (where *CT* is threshold cycle) method. Primers were as follows:

TNF-α-F: 5′-TGTCTCAGCCTCTTCTCATTCC-3′,

TNF-α-R: 5′-TTAGCCCACTTCTTTCCCTCAC-3′;

CCL-5-F: 5′-TGCCCACGTCAAGGAGTATTTC-3′,

CCL-5-R: 5′-AACCCACTTCTTCTCTGGGTTG-3′;

IL-1β-F: 5′-AACCTGCTGGTGTGTGACGTTC-3′,

IL-1β-R: 5′-CAGCACGAGGCTTTTTTGTTGT-3′;

CCL-2-F: 5′-CGGCGAGATCAGAACCTACAAC-3′,

CCL-2-R: 5′-GGCACTGTCACACTGGTCACTC-3′;

β-actin-F: 5′-CACTGCCGCATCCTCTTCCTCCC-3′,

β-actin-R: 5′-CAATAGTGATGACCTGGCCGT-3′.

### Immunoblotting

Cell pellets or mice brain tissue were lysed in radioimmunoprecipitation assay buffer (Sigma) containing phosphatase (PhosSTOP, Roche) and protease inhibitors (Complete Tablets, Roche). Protein concentrations were measured using a BCA (bicinchoninic acid) Protein Assay Kit (Thermo Fisher Scientific). Equal protein quantities were electrophoresed and transferred onto a nitrocellulose membrane using a Mini Trans-Blot Cell (Bio-Rad). Membranes were then blocked by incubating in blocking buffer (tris-buffered saline with 0.5% Tween 20 and 5% BSA) for 1 h and probed with relevant primary antibodies. After washing, membranes were incubated with the appropriate secondary antibodies. The blots were detected using enhanced chemiluminescent (ECL) reagent (Thermo Fisher Scientific). The following antibodies were used: mouse monoclonal anti-JEV NS5 (1 ng/ml for Western blot assay, prepared by our laboratory), rabbit polyclonal anti-phosphor-NR1-Ser890 (Cell Signaling Technology), rabbit polyclonal anti-phosphor-NR2-Y1472 (ABclonal Technology), rabbit polyclonal anti-caspase-3 (Proteintech), mouse monoclonal anti-glyceraldehyde-3-phosphate dehydrogenase (GAPDH) (ABclonal Technology), rabbit polyclonal anti-β-tubulin (ABclonal Technology), and horseradish peroxidase-conjugated anti-mouse and anti-rabbit secondary antibodies (Boster). Appropriate concentrations for these commercial antibodies were used following the manufacturer’s guidelines.

### Intracellular Ca^2+^ staining

Primary mouse neuron/glial cells were seeded in 12-well plates at a density of 10^5^ cells per well and maintained as aforementioned. Fluo 4/Am (Molecular Probe) was then added at a final concentration of 5 μmol/L, and the treated cells were further incubated at 37 °C with 5% CO_2_ for 30 min. The treated cells were removed, centrifuged for 5 min at 2000 rpm, washed with Ca^2+^-free PBS twice to remove the excess dye, and finally resuspended to 0.5 ml with Ca^2+^-free PBS. Fluorescence signals were collected and analyzed using flow cytometry. The intracellular Ca^2+^ concentration was quantified as mean fluorescence intensity (MFI).

### Annexin V-FITC/PI staining

Cells were trypsinized and washed with serum-containing medium. The samples (10^6^ cells) were centrifuged for 5 min at 2000 rpm and the cell supernatants were discarded. The cells were then stained using an Annexin V-FITC/PI Kit (4A Biotech) in accordance with the manufacturer’s instructions. The number of apoptotic cells was detected and analyzed using flow cytometry. The cells were divided into four sections: Q1: Annexin V-FITC− PI+, was representative of necrotic and mechanically damaged cells; Q2: Annexin V-FITC+ PI+, was representative of late apoptotic cells; Q3: Annexin V-FITC+ PI−, was representative of early apoptotic cells; and Q4: Annexin V-FITC− PI−, was representative of living cells.

### Caspase-3 activation assay

The enzymatic activity of caspase-3 was determined by using Caspase-3 Colorimetric Assay Kit (KeyGEN) according to the manufacturer’s instructions.

### Hematoxylin and eosin (H&E) staining, immunohistochemistry (IHC), and terminal deoxynucleotidyl transferase-mediated dUTP nick end labelling (TUNEL) assay

Treated mice were anesthetized with ketamine-xylazine (0.1 ml per 10 g of body weight), and brain tissues were collected and embedded in paraffin for coronal sections. The sections were used for H&E staining, IHC, and TUNEL assay. For IHC, brain sections were incubated overnight at 4 °C with primary antibodies against glial fibrillary acidic protein (GFAP) (Dako), ionized calcium binding adapter molecule 1 (IBA-1) (Wako), or neuronal nuclei (NeuN) (Chemicon). After being washed, slides were incubated with appropriate secondary antibodies and washed, and protected by the cover. For TUNEL assay, an In Situ Cell Death Detection Kit (Roche) was used according to the manufacturer’s instructions.

### Plaque assay

Virus loads in cell culture supernatants and mice brain tissues were assessed by plaque assay in BHK-21 cells as described in our previous study [26]. The visible plaques were counted, and viral titers were calculated. Results were measured as PFU per gram of tissue weight or milliliter of the supernatant.

### Statistical analysis

All experiments were carried out at least three times with similar conditions. Analyses were conducted using GraphPad Prism, version 5 (GraphPad Software, San Diego, USA). Results are expressed as the mean ± standard error (SEM), except for viral loads, which are expressed as the median. Statistical differences among the experimental groups were determined using two-way analysis of variance (ANOVA) with subsequent *t* tests using the Bonferroni posttest used for multiple comparisons. For all tests, a *p* values of < 0.05 was considered significant.

## Results

### JEV infection activates NMDAR by enhancing the phosphorylation of NMDAR subunits

**Fig. 1:**
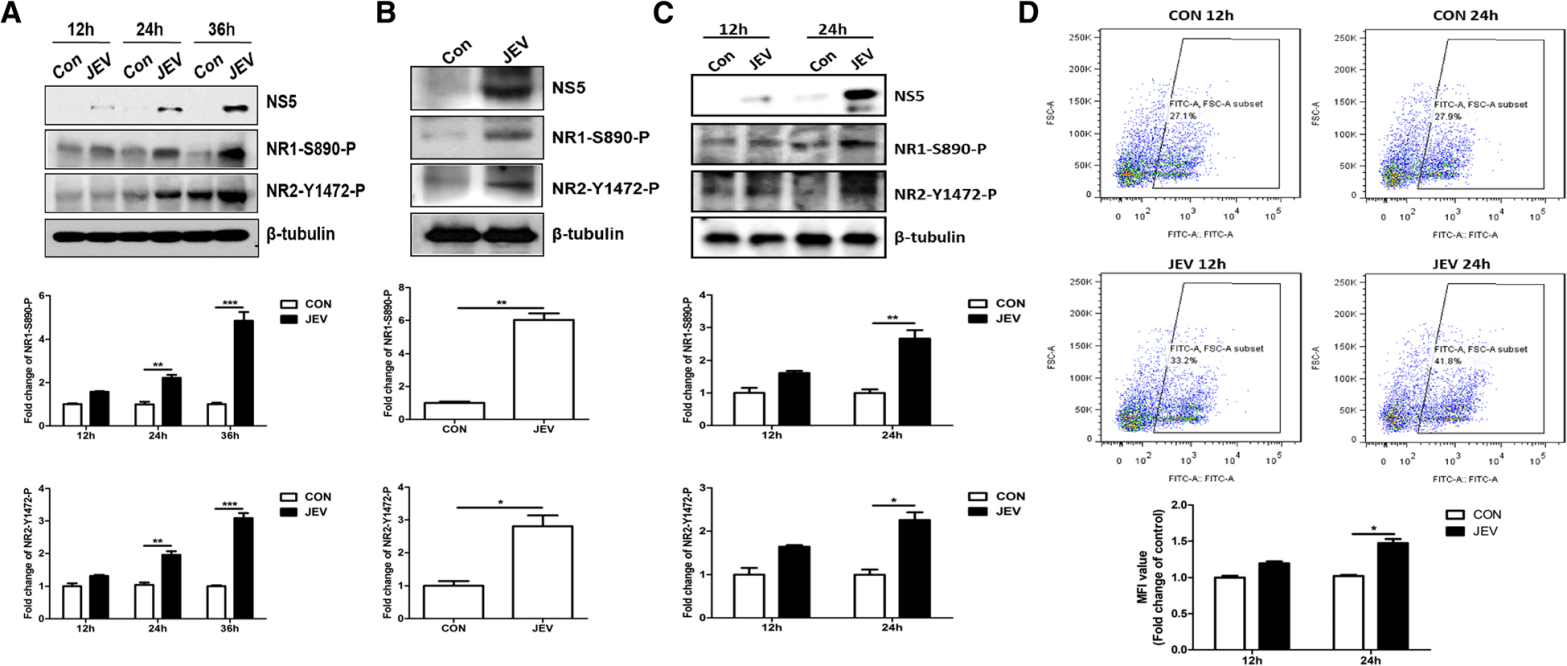
JEV infection stimulates the activity of NMDAR. **a** N2a cells and **c**–**d** primary mouse neuron/glia cultures were infected with JEV at an MOI of 1, and samples were collected at indicated time points. **b** Mice were inoculated intraperitoneally with DMEM or 10^6^ PFU JEV, and brain tissues were collected and lysed on day 6 post-infection. The extracts from cultured cells and mice brain tissues were either subjected to Western blot analysis (**a**–**c**) or to flow cytometry analysis (**d**). The lower panels represent the quantification of data corresponding to **a**–**d**. Protein levels were quantified by immunoblot scanning and normalized to the amount of β-tubulin. The relative intracellular calcium concentration was representative with mean fluorescence intensity (MFI) and normalized to the control. Data are expressed as means ± SEM from three independent experiments. *P < 0.05, **P < 0.01, ***P < 0.001

NMDAR is a tetrameric complex composed of two obligatory GluN1 (or NR1) and two modulatory GluN2 (or NR2) subunits. Phosphorylation of amino acid sites on different subunits of NMDAR plays a vital role in regulating the NMDAR activity [27–29]. To determine the effect of JEV infection on NMDAR activation, we examined the phosphorylation levels of NMDAR subunits NR1 and NR2B in JEV-infected cultured cells and mice brain tissues. The results revealed that JEV infection significantly increased the activity of NMDAR by enhancing the phosphorylation levels of NMDAR subunits in N2a cells (mouse neuronal cell line) and mice brain tissues in a time-dependent manner (Fig. 1a, b). The JEV-induced hyperphosphorylation of NMDAR subunits was further analyzed in primary mouse neuron/glia cultures infected with JEV. As shown in Fig. 1c, the results were concordant with those observed in JEV-infected N2a cells and mice brain tissues. Since NMDAR activation is associated with an increased influx of Ca^2+^ [13, 14], we next determined the effect of JEV infection on the mobility of Ca^2+^ in primary mouse neuron/glial cells. As shown in Fig. 1d, JEV infection triggered the influx of Ca^2+^ in a time-dependent manner; these results further demonstrate that JEV infection stimulates the NMDAR activity.

### NMDAR blockade prevents JEV-induced neuronal cell death and reduces the production of glutamate and inflammatory cytokines in vitro

**Fig. 2:**
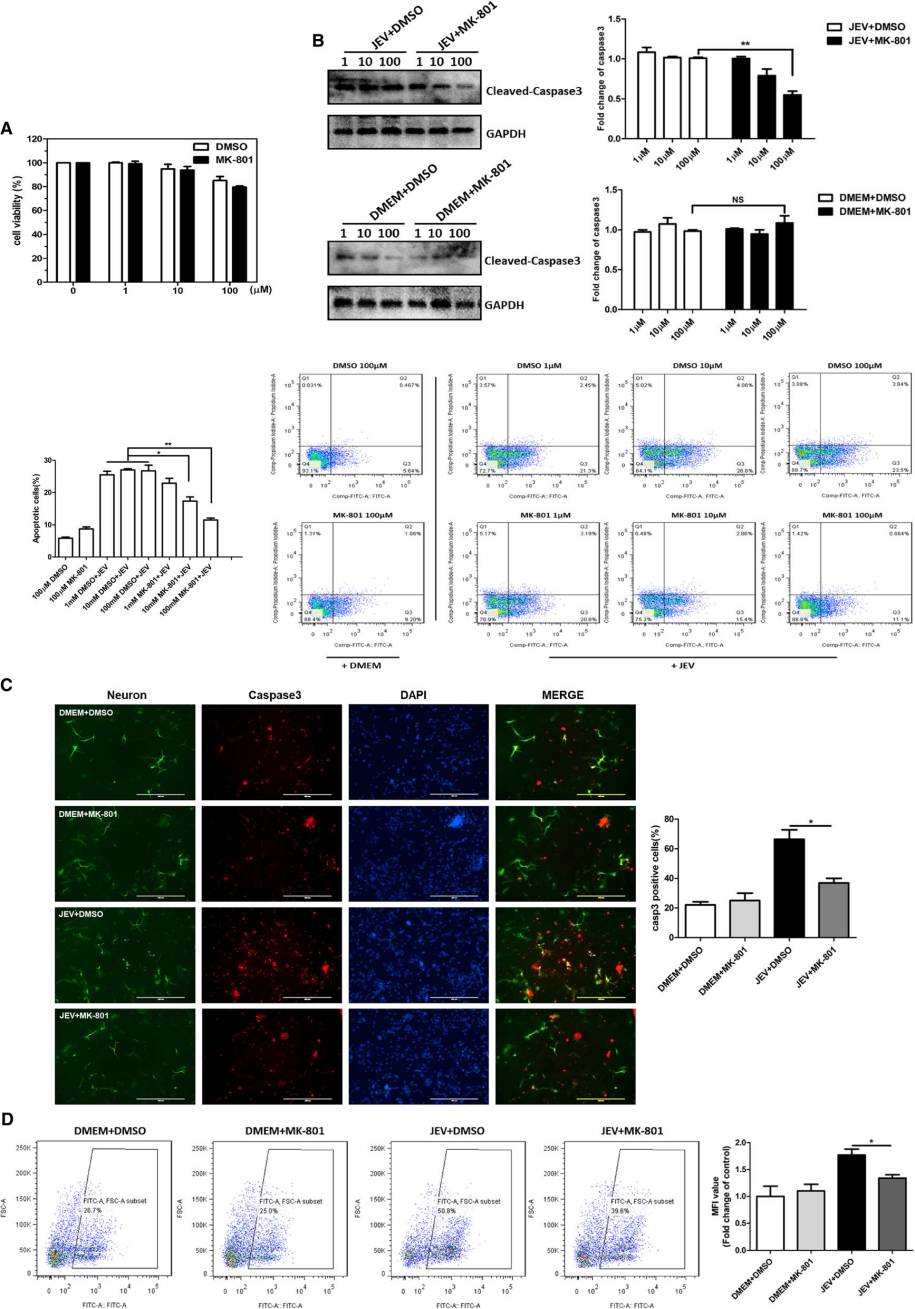
Blockade of NMDAR by MK-801 prevents JEV-induced neuronal cell death in vitro. **a** Cell viability assay at indicated concentrations of MK-801. **b** The analysis of apoptotic markers caspase-3 and phosphatidylserine in primary mouse neuron/glia cells. The cells were either mock-infected or infected with JEV at an MOI of 1. Subsequently, cells were treated with indicated concentrations of MK-801 or DMSO after infection and every 24 h post-infection for 2 days. Caspase-3 protein levels (upper panel) and phosphatidylserine localization (lower panel) were determined by immunoblotting and flow cytometry, respectively. Protein levels were quantified by immunoblot scanning and normalized to the amount of GAPDH levels. **c** Immunofluorescence staining of neuronal nuclei marker NeuN (green) and apoptosis marker caspase-3 (red). Primary mouse neuron/glia cells were subjected to infection and treatment paradigm (10 μM concentrations of MK-801) as described in **b**, and cells were stained with corresponding antibodies. The right panel shows the quantification of caspase-3-positive cells. Scale bar, 400 μm. **d** Measurement of intracellular Ca^2+^ concentration. Primary mouse neuron/glia cells were subjected to infection and treatment paradigm (10 μM concentrations of MK-801) as described in **b**. Subsequently, cells were treated with Fluo 4/Am and collected for analysis with flow cytometry. Data are expressed as means ± SEM from three independent experiments. *P < 0.05, **P < 0.01

It has been well-established that the activated NMDAR triggers and exacerbates the excitatory neuronal injury [30–32]. Considering our confirmed analysis of NMDAR activation by JEV infection (Fig. 1a–d), we speculated that blocking the NMDAR activity may cause a reduction in JEV-mediated neuronal injury. To test our hypothesis, a high-affinity NMDAR antagonist MK-801 was employed in a series of in vitro experiments using mouse primary neuron/glia cultures. First, we examined the effect of MK-801 treatment on the viability of cultured cells by employing a luminescence-based cell viability assay. This assay determines the number of viable cells in culture based on the quantification of ATP present, which indicates the presence of metabolically active cells. The results revealed that MK-801 exhibited no cytotoxic effect under all experimental concentrations tested (Fig. 2a). To evaluate the effect of MK-801 in preventing neuronal cell death during JEV infection, mock- or JEV-infected cells were treated with MK-801 or DMSO after infection (virus absorption) and at every 24 h post-absorption for 2 days. The activation of apoptosis in these samples was determined by measuring the levels of caspase-3 and phosphatidylserine, which are widely known markers of apoptosis. Our immunoblot analysis and Annexin V-FITC/PI staining revealed that MK-801 prevented the JEV-induced neuronal cell apoptosis in a dose-dependent manner and that medium concentration of MK-801 (10 μM) can reduce the cell death efficiently (Fig. 2b). The immunofluorescence analysis of MK-801- or DMSO-treated primary cultured cells showed the similar results as observed in immunoblot analysis (Fig. 2c). Furthermore, the treatment of infected cells with MK-801 reduced the JEV-triggered influx of Ca^2+^ significantly (Fig. 2d), suggesting that blockade of NMDAR can halt the JEV-induced neuronal cell death.

### NMDAR blockade reduces the production of glutamate and inflammatory cytokines in vitro

**Fig. 3:**
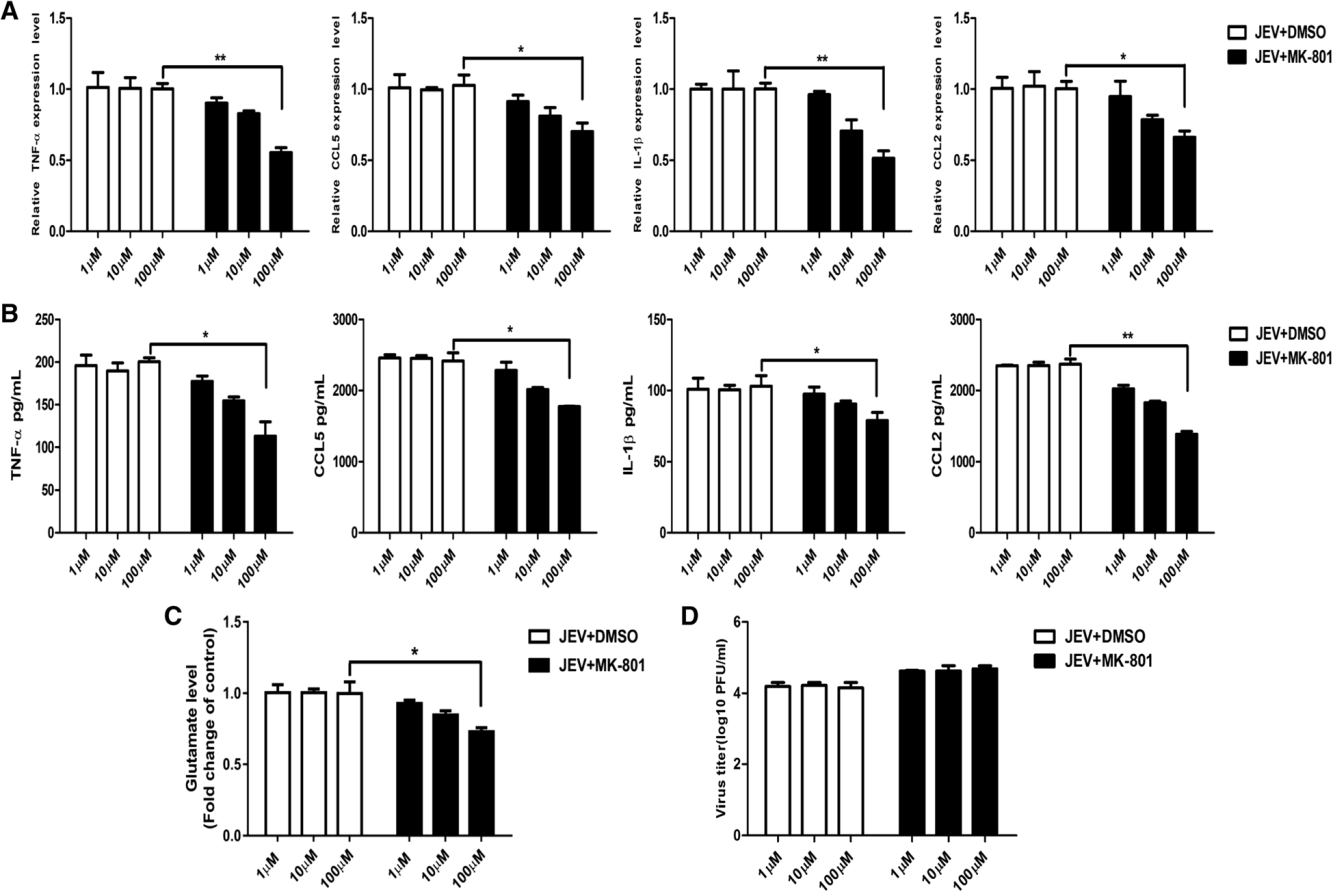
MK-801-mediated blockade of NMDAR reduces JEV-triggered glutamate and inflammatory cytokines production in vitro*.* **a**–**d** Primary mouse neuron/glia cells were subjected to infection and treatment paradigm as described in Fig. 2b. The mRNA (**a**) and protein (**b**) levels of TNF-α, CCL-5, IL-1β, and CCL-2 were analyzed by quantitative real-time PCR and ELISA, respectively. The glutamate protein levels (**c**) were determined by ELISA. Viral titers (**d**) in cell supernatants were determined by plaque assay. Data are expressed as means ± SEM from three independent experiments. *P < 0.05, **P < 0.01

Since the substances released by dead neuronal cells can activate glial cells to produce glutamate and inflammatory cytokines [33], we next measured the levels of glutamate and inflammatory cytokines (TNF-α, IL-1β, CCL5, and CCL2) in the culture media. As shown in Fig. 3a–c, JEV infection triggered the excessive release of glutamate and inflammatory cytokines, whereas the treatment of infected cells with MK-801 significantly repressed the production of glutamate and inflammatory cytokines in a dose-dependent manner. Furthermore, we found that MK-801 treatment did not interfere with the ability of the virus to replicate in the cultured cells as the viral titers were similar to those in control cells (Fig. 3d). Taken together, these results demonstrate that the blockade of NMDAR by MK-801 prevents JEV-induced neuronal cell death and reduces the production of glutamate and inflammatory cytokines in vitro, suggesting the critical role of NMDAR activation in JEV-induced neuronal death.

### NMDAR blockade abrogates JEV-induced neuronal cell death and glial cell activation in mice brain tissues

**Fig. 4:**
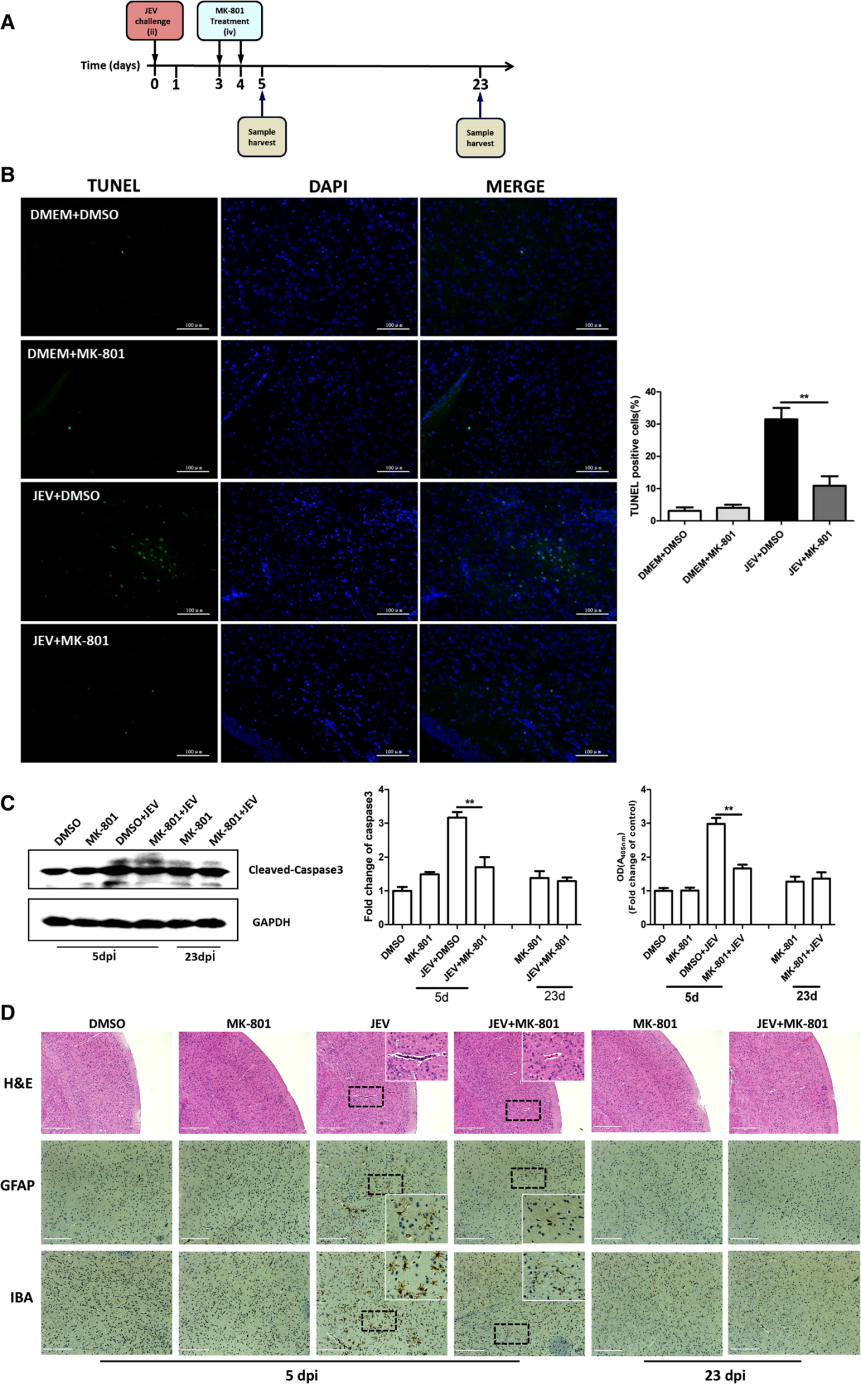
NMDAR blockade by MK-801 abrogates JEV-caused neuronal death and glia activation in mouse brain. **a** Diagram of in vivo experiment model. Mice were subjected to JEV infection and MK-801 treatment paradigm as described in the methods section. **b** The apoptotic neurons (green) in the mouse brain on day 5 after JEV infection were detected using the TUNEL Assay Kit. The right panel shows the quantification of TUNEL-positive cells. Scale bar, 100 μm. **c** Caspase-3 protein levels (upper panel) and enzymatic activity (lower panel) were determined by Western blot and Caspase-3 Colorimetric Assay Kit. Protein levels were quantified by immunoblot scanning and normalized to the amount of GAPDH expression. The enzymatic activity is presented as optical density values and is normalized to the control. **d** H&E staining and IHC to observe the pathological changes and activation of glial cells. White box visualization indicates perivascular cuffing and activated glia cells. Scale bar, 400 μm. Figures are the representative of three mice with similar results. **P < 0.01

To delineate the significance of NMDAR activation in JEV-caused encephalitis in vivo, a mouse model of JEV infection was established (Fig. 4a). MK-801 or DMSO was administered intravenously into mock- or JEV-infected mice on days 3 and 4 post-infection to inhibit the activity of NMDAR. On days 5 and 23 post-infection, the mice were sacrificed and brain samples were collected and processed for subsequent experiments. To examine whether MK-801 treatment can reduce neuronal cell damage, brain samples processed on day 5 post-infection, were subjected to a TUNEL assay. The numbers of TUNEL-positive cells were found to be significantly lower with MK-801 treatment in JEV-infected mice than in vehicle-treated infected mice (Fig. 4b). Furthermore, we evaluated the cell apoptosis by examining the expression and enzymatic activity of caspase-3 in the brain samples collected on day 5 post-infection. Similar to our in vitro findings, the treatment of JEV-infected mice with MK-801 caused a significant reduction in expression and activity of caspase-3 compared to control infected mice (Fig. 4c).

The analysis of histopathological changes by H&E staining of cerebrum showed the signatures of meningitis and perivascular cuffing in JEV-infected mice at 5 days after infection, whereas these indicators of encephalitis were reduced in JEV-infected mice receiving MK-801 treatment (Fig. 4d). The surviving JEV-infected+MK-801-treated mice exhibited normal histological features in the brain at 23 days post-infection (Fig. 4d). In order to interrogate the effect of MK-801 treatment on JEV-induced gliosis and neuronal death, the mice brain sections were subjected to IHC staining using the antibodies that can recognize the astrocyte marker protein GFAP and the microglia marker protein IBA-1. An aberrant increase in the number of activated microglia and astrocytes was detected at day 5 post-infection, whereas MK-801 treatment reduced the reactive microgliosis and astrogliosis in infected mice (Fig. 4d). These results showed that inhibition of NMDAR activity attenuates the neuronal cell death and ameliorates pathological changes in mice brain during JEV infection.

### NMDAR blockade reduces JEV-induced glutamate release and inflammatory response in mice brain tissues

**Fig. 5:**
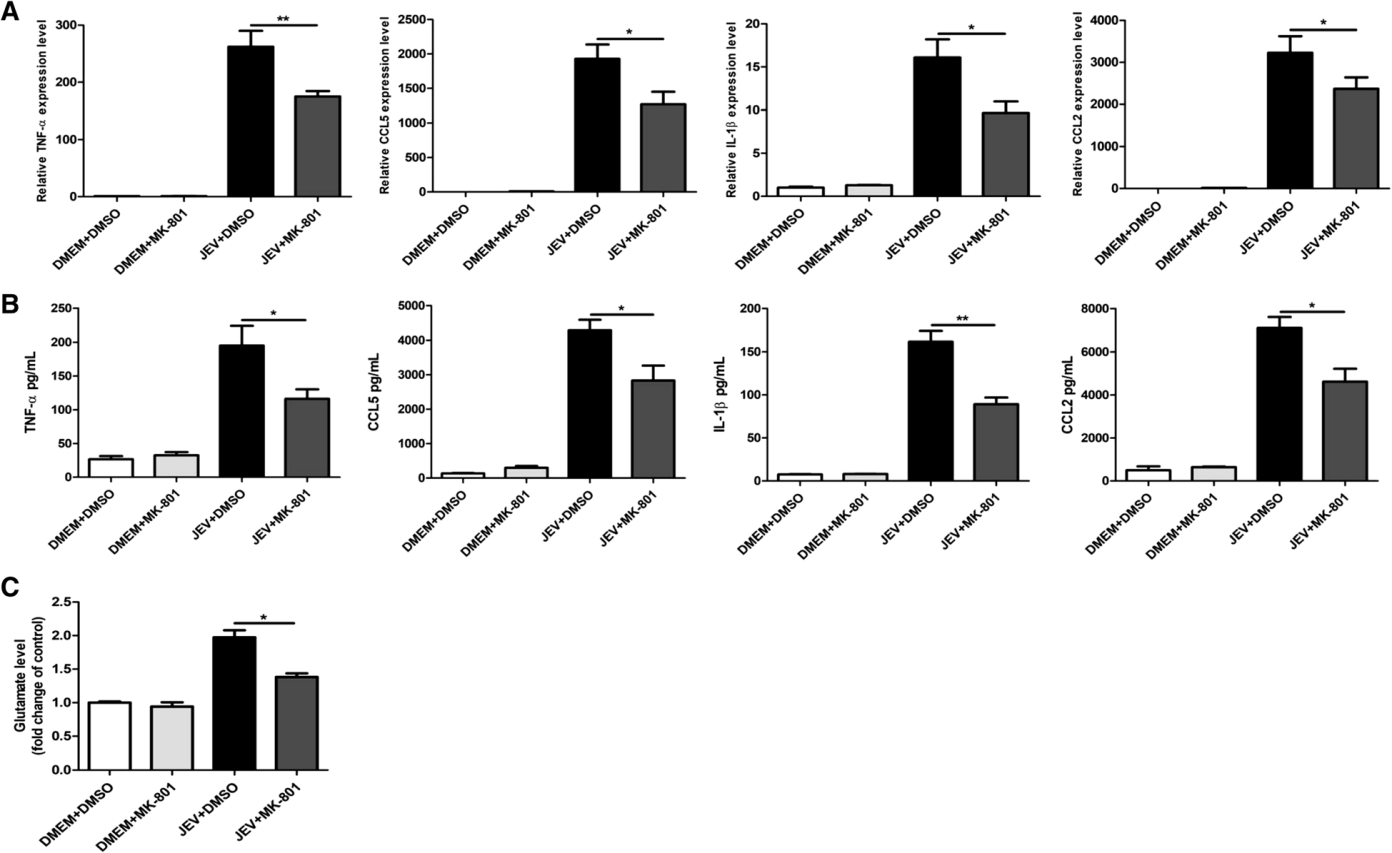
NMDAR blockade by MK-801 suppresses JEV-triggered glutamate release and inflammatory response in mouse brain. Mice were subjected to JEV infection and MK-801 treatment paradigm as described in Fig. 4a. Brain samples were collected on day 5 after JEV infection. **a**, **b** The mRNA (**a**) and protein (**b**) levels of TNF-α, CCL-5, IL-1β, and CCL-2 in brain lysates were analyzed by quantitative real-time PCR and ELISA, respectively. **c** The glutamate protein levels in brain lysates were determined by ELISA. Similar results were obtained in three mice. *P < 0.05, **P < 0.01

Since the NMDAR blockade attenuated the reactive microgliosis and astrogliosis in our mouse model of JEV infection, we next assessed the effect of NMDAR blockage on the abundance of glutamate and inflammatory cytokines (TNF-α, IL-1β, CCL5, and CCL2) in the mouse brain. As shown in Fig. 5a–c, JEV infection triggered the release of a large amount of glutamate and inflammatory cytokines, whereas MK-801 treatment markedly decreased the JEV-triggered glutamate and cytokine production in mice brain. These findings indicate that NMDAR blockade can reduce glutamate release and suppress inflammatory response induced by JEV infection in mouse brain.

### NMDAR blockade reduces mouse lethality induced by JEV infection

**Fig. 6:**
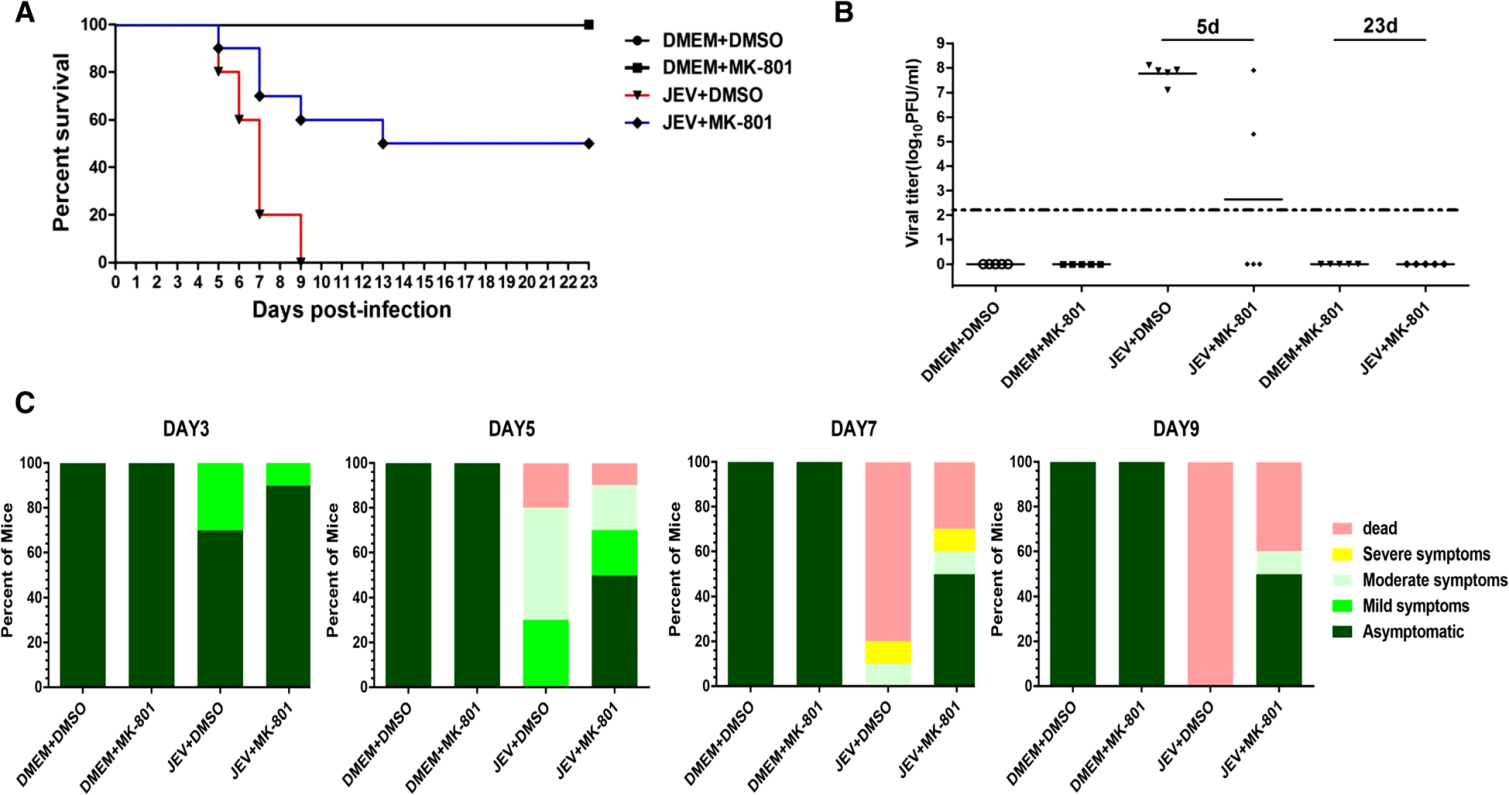
MK-801 treatment reduces lethality in JEV-infected mouse model. Mice were subjected to JEV infection and MK-801 treatment paradigm as described in Fig. 4a. **a** Survival of mice in each group was monitored for 23 days after JEV inoculation. Data were collected and shown as Kaplan-Meier survival curves. *n* = 10 mice. **b** Viral titers in the mouse brain on days 5 and 23 after JEV infection were determined by plaque assay. The viral titers are shown as log_10_ PFU/ml. *n* = 5 mice. **c** Presentation of clinical signs of disease in mice on indicated days following JEV infection. *n* = 10 mice

Given the role of NMDAR blockade in preventing the JEV-triggered neuronal apoptosis and inflammatory response both in vitro and in vivo, we assessed the potential of NMDAR blocker in protecting mice against JEV-associated lethality. All mice in the MK-801- or DMSO-treated group survived during the observation. Mice in the JEV-infected group, treated with vehicle or MK-801, started to display morbidity and mortality on day 5 post-infection. A high mortality rate (100%) was observed in the vehicle-treated mice on days 5 to 9 post-infection. In contrast, administration of MK-801 protected mice from JEV-induced lethality within 5 to 9 days post-infection, and a survival rate of 50% was recorded during the entire 23-day period of observation (Fig. 6a). Furthermore, we determined the viral loads in infected mice brain tissues by measuring the viral titers through plaque assay. Brain homogenates prepared on day 5 post-infection from JEV-infected+MK-801-treated mice showed a significant reduction in viral titers compared with that from untreated infected mice (Fig. 6b).

To substantiate the effect of NMDAR blockade on neural development and brain function, we scored the behavioral signs observed in all the experimental mice. As expected, treatment with MK-801 caused improved behavioral signs in JEV-infected mice. The non-infected mice belonging to MK-801 and DMSO control groups showed no obvious clinical symptoms during the observation (Fig. 6c). Thus, these results suggest that blockade of NMDAR can provide a protective effect against JEV-induced mortality.

## Discussion

In the present study, we showed that activation of neuronal NMDAR by JEV is indispensable to JEV-mediated neuronal cell death. We evaluated the neuroprotective effect of NMDAR blockade against JEV-associated neuronal apoptosis and neuroinflammation. It was found that the blockade of NMDAR by its antagonist MK-801 can prevent JEV-induced neuronal cell death and production of glutamate and inflammatory cytokines both in vitro and in vivo, which eventually provides protection against acute encephalitis and reduces mortality in a mouse model of JEV infection.

NMDARs mediate most excitatory neuronal transmission in the CNS and play pivotal roles in the regulation of synaptic activity. The activation of NMDAR results in hyperphosphorylation of NMDAR subunits and an increased influx of Ca^2+^ that are considered to underlie the basic mechanisms leading to neuronal injury [13, 34]. The dysfunction of these receptors contributes to the progression of several psychiatric and neurological disorders such as Alzheimer’s disease, cerebral ischemia, and Parkinson’s disease [35–37]. However, the role of NMDAR in JEV-mediated neuronal apoptosis has not been yet explored. Herein, we found that JEV infection also promotes NMDAR activity by augmenting the phosphorylation of NMDAR subunits (NR1 and NR2) and by triggering the influx of Ca^2+^. Using the NMDAR-specific antagonist MK-801, we confirmed the role of NMDAR in JEV-mediated neuronal injury. However, the precise molecular mechanism underlying the JEV-induced NMDAR activation remains unclear in this study.

There were many kinds of NMDAR antagonists, while memantine and MK-801 were the two noncompetitive antagonists which had been used most among them. In this study, we chose MK-801 as a representative to antagonize NMDAR activity, for it has high affinity to NMDAR. MK-801, also named dizocilpine, has been shown to exert a reliable neuroprotective effect in several neurodegenerative disease models. For instance, MK-801 treatment served as a valuable tool to enhance hippocampal neurogenesis in a rat model of Parkinson’s disease [36]. In rat cerebral ischemia models, whether alone or in combination, MK-801 treatment displayed an effective function in protecting neurons from being dead [35]. Other studies have shown that the apoptotic neurons which suffered from JEV infection have their ability to release inflammatory cytokines which subsequently trigger the neuroinflammation and glutamate release [4, 8, 38]. Given the neuroprotective role of MK-801 in neurodegenerative diseases, the involvement of apoptotic neurons in the inordinate production of glutamate and inflammatory cytokines, and our confirmed analysis of increased NMDAR activity upon JEV infection, we hypothesized that MK-801-mediated blockade of NMDAR may provide protection against neuronal cell death and suppress undue inflammatory response during JEV infection. In this regard, a series of in vitro and in vivo experiments were conducted using the primary mouse neuron/glia cultures and a mouse model of JEV infection. Our results demonstrated that MK-801 treatment of JEV-infected primary cultured cells and JEV-challenged mice prevents the neuronal apoptosis and abrogates the production of glutamate and inflammatory cytokines in these cultured cells and mice brain tissues. Moreover, the administration of MK-801 protected the mice from mortality (50% survival rate) and caused improved behavioral and clinical signs in JEV-infected mice. These findings suggest that the reduction of JEV-induced neuronal cell death and inflammatory response by blocking the activity of NMDAR can potentially attenuate the progression of events leading to morbidity and mortality caused by JEV infection and, thus, seems to be a remedy against JE. Since we observed that MK-801 treatment extenuates neuroinflammation induced by JEV infection, we surmised that this phenomenon might be associated with an indirect effect of NMDAR blockade. The possibility whether NMDAR can directly participate in neuroinflammatory signaling pathways or not requires further investigations.

Albeit NMDAR antagonists are frequently reported to be used in treating neurodegeneration diseases, people are still concerned about their clinical applications. The reason for this concern is that NMDAR is a major excitatory receptor in the mammalian brain; therefore, blockade of this receptor could cause a series of deleterious effects owing to fluctuations in neurotransmission [39, 40]. For instance, some studies reported that a single high-dose or low-dose repeated administration of MK-801 may cause schizophrenia in association with cognitive dysfunction in animal models [41–43]. However, in our study, we did not observe any abnormal behaviors like schizophrenia in the MK-801-single-treated mice. These differences may attribute to administration mode, dose, and animal species.

There have already been many reports on the therapeutic drug candidates for JEV infection. These candidates exert antiviral effects through different strategies: by directly inhibiting the JEV replication (minocycline, nitazoxanide, and quercetin) [44–46] or by targeting JEV-induced inflammatory signaling pathways (etanercept and small RNA inhibitors) [24, 25, 47]. Herein, we used NMDAR antagonist to cure encephalitis associated with JEV infection. To the best of our knowledge, it is the first report demonstrating the use of a drug targeting the neuronal cells to treat JE. However, none of these drugs can completely cure the mice suffered from the lethal viral infection. Future studies are needed to be established to evaluate whether combinations of drugs can provide the best therapeutic effects.

## Conclusions

This study provides evidence that NMDAR plays a critical role in JEV-induced neuronal cell damage and inflammatory response. Our in vitro and in vivo functional studies revealed that NMDAR blockade provides effective protection against neurodegeneration and neuroinflammation occurred during the JEV infection. The observed protective effect is identified to be associated with reduced neuronal apoptosis, suppressed glutamate and inflammatory cytokine production, and decreased mortality of mice. Therefore, NMDAR can be considered as a potential therapeutic target to treat JE.

## Abbreviations

BSA: Bovine serum albumin
CCL2: Monocyte chemotactic protein 1
CCL5: Chemokine (C-C motif) ligand 5
CNS: Central nervous system
DAPI: 4′,6-Diamidino-2-phenylindole
ELISA: Enzyme-linked immunosorbent assay
GAPDH: Glyceraldehyde-3-phosphate dehydrogenase
GFAP: Glial fibrillary acidic protein
IBA-1: Ionized calcium binding adapter molecule 1
IL-1β: Interleukin-1β
JE: Japanese encephalitis
JEV: Japanese encephalitis virus
NeuN: Neuronal nuclei
NMDAR: *N*-Methyl-d-aspartate receptor
TNF-α: Tumor necrosis factor–α
TUNEL: Terminal deoxynucleotidyl transferase–mediated deoxyuridine triphosphate nick end labeling
